# Validity and Reliability of Intraoral and Plaster Models’ Photographs in the Assessment of Little's Irregularity Index, Tooth Size-Arch Length Discrepancy, and Bolton's Analysis

**DOI:** 10.7759/cureus.23067

**Published:** 2022-03-11

**Authors:** Wael A Alrasheed, Amer M Owayda, Mohammad Y Hajeer, Tarek Z. Khattab, Wael H Almahdi

**Affiliations:** 1 Department of Orthodontics, Faculty of Dentistry, University of Damascus, Damascus, SYR; 2 Department of Orthodontics, Faculty of Dentistry, University of Hama, Hama, SYR; 3 Department of Periodontics, Faculty of Dentistry, University of Damascus, Damascus, SYR

**Keywords:** plaster models, reporoducibility, accuracy, inter-observer reliability, intra-observer reliability, calibration, bolton’s analysis, tooth-size-arch-length discrepancy, little's irregularity index, intraoral photographs

## Abstract

Background

Dental impressions have been required to obtain proper study models. This procedure is time- and labor-consuming for the orthodontist and could be exhausting to the patient, especially when braces are fitted in the context of a research project. This study aimed to assess the accuracy, reliability, and reproducibility of using intraoral photographs and plaster models’ photographs in measuring Little's Irregularity Index (LII), tooth size-arch length discrepancy (TSALD), and Bolton's ratios.

Methods

A total of 52 dental arches of 26 patients were included in this study. Plaster models, occlusal intraoral photographs, and photographs of the collected plaster models were obtained for each patient. Then, LII, TSALD, and Bolton’s ratios were measured using a manual caliper for plaster models’ measurements and a software-based on-screen method for the photographs.

Results

The intraclass correlation coefficients (ICCs) of measurements made on intraoral photographs and photographs of plaster models were high (ranging from 0.90 to 0.99 and from 0.88 to 0.99, respectively), indicating a high level of agreement with the gold standard measurements. In addition, the differences were insignificant. The intra-/inter-examiner ICCs ranged from 0.90 to 0.99/0.92 to 0.99 and from 0.85 to 0.99/0.88 to 0.98 for plaster models and intraoral photographs of the dental arches, respectively. The analysis of reproducibility of capturing intraoral photographs of the dental arches on two different occasions showed high ICCs ranging from 0.96 to 0.99 with almost no significant differences between repeated measurements (P > 0.05).

Conclusion

LII, TSALD, and Bolton’s overall and partial ratios can be measured from intraoral photographs of the dental arches with high accuracy, reliability, and reproducibility. Therefore, this methodology can be suggested for use in research projects when multiple records of the dental arches are required instead of depending on time- and labor-consuming procedures of ordinary dental impressions.

## Introduction

The general trend in the orthodontic practice is to become digital in many aspects [1]. Since the 1980s, digital photographs have been available and play a principal role in the orthodontic practice for documentation and diagnosis purposes [2]. Now photographs have an important role in teaching, scientific research, and medical examination [3].

Successful orthodontic treatment is based on a comprehensive diagnosis and treatment planning. A few of the fundamental factors in the diagnosis are the spacing condition, teeth size, arch form, and dimensions, as well as the tooth-arch discrepancies [4]. The major role of intraoral photographs is to enable orthodontists to document and analyze the occlusal relationships as well as the dental and soft-tissue features to arrive at a good diagnosis and appropriate treatment planning [2].

In 1975, Robert Little developed Little’s Irregularity Index (LII). The index was proposed to assess teeth irregularity, crowding, relapse, and alignment of the anterior teeth as it measures the linear displacements in the horizontal plane between contact points of anterior teeth, ignoring vertical displacement, from the mesial surface of one canine to the contralateral one. The sum of the five liner measurements of displacements was the LII score. The higher the index value, the more the severe irregularity of the teeth was [5]. LII has been originally developed for mandibular incisors to study relapse; however, researchers have used it to assess upper and lower incisors irregularity [6,7].

Tooth size-arch length discrepancy (TSALD) is widely used in study models to assess the level of harmony between tooth size and the supporting basal bone [8]. Bolton analysis is another important measurement used to identify disharmony between maxillary and mandibular tooth size, which is considered an important factor to ensure the success of orthodontic treatment. With the application of the suggested formulas, the overall ratio should be 91.3% (±1.91) and the partial (anterior) ratio should be 77.2% (±1.65) [9].

Assessment of the LII scores, TSALD, and Bolton analysis has been performed by the majority of previous studies using plaster models and calipers in a direct manner [5,6]. Others have measured previous variables indirectly using two-dimensional (2D) and three-dimensional (3D) methods. 2D methods have included 2D scans of plaster models or 2D images of the occlusal views. The obtained images have been then analyzed either manually [10] or on-screen using dedicated software [11,12]. 3D methods have included digital models and have been used by several researchers [1,4,13-19]. Despite the wealth of knowledge in 3D imaging, it requires the use of 3D imaging techniques with the resultant additional costs, time, and labor.

Many studies have used 2D digital images of poured plaster models to perform software-based measurements of tooth movement during active treatment but these studies have not reported the accuracy and reproducibility of their methods [20-22]. Dental impressions have been required to obtain proper study models. This procedure is time- and labor-consuming for the orthodontist and could be exhausting to the patient. When braces are fitted, impression taking becomes a difficult task and if several impressions are required in the context of a research project, this would impose an additional burden on patients and researchers. Therefore, taking intraoral images of dental arches instead of impressions seems to be a very convenient alternative.

The validity and reliability of measurements made on photographs of study models have been evaluated in previous reports [23,24]. However, it seems that there is only one paper in the literature with the aim of validating the use of intraoral images of the dental arches for performing dental measurement [25]. They found that the analysis of LII using photographs was valid. However, the practicability of their suggested method of imaging was questionable and their evaluation was only confined to the LII, which is not the only variable that is used in our ordinary plaster model analysis.

Therefore, the primary aim of the current work was to evaluate the validity, reliability, and repeatability of measurements made directly on intraoral photographs compared to those made on poured plaster models in the assessment of the LII, TSALD, and Bolton ratios. As a secondary aim, the comparisons were also accomplished with those measurements made on photographs taken of the corresponding poured plaster models.

## Materials and methods

This trial was registered at ClinicalTrials.gov (identifier: NCT03648515). After obtaining the ethical approval from the Local Research Ethics Committee at the University of Damascus, 143 patients who seek orthodontic treatment at the Department of Orthodontics, Dental School were examined during January, February, and March in 2018. A preprint of the manuscript was posted on the Research Square platform on January 17, 2020, with the following DOI: 10.21203/rs.2.21031/v1. Of patients, 26 were randomly selected with the following inclusion criteria: complete permanent dentition without any big carious lesions or fixed prosthetic or shape and size disturbances (regardless of third molars) associated with upper and lower crowded arches. Upper and lower orthodontic models were obtained from “Additional Silicone” impressions (Zetaplus, Zhermack Dental, Badia Polesine, Italy) for each patient and then poured with dental plaster (Elite Ortho Type III, Zhermack Dental, Badia Polesine, Italy).

Occlusal intraoral photographs were taken with a digital single-lens reflex (DSLR) camera (EOS 5D Mark III, Canon, Tokyo, Japan) with a dedicated lens (Canon EF 100mm f/2.8L Macro IS USM) and a ring flash (Yongnuo YN-14EX TTL macro ring light flash). Photographs were taken at two occasions with an interval of two weeks by two third-year MSc students (A.O. and W.R.) at the Department of Orthodontics using the following settings for intraoral photographing: manual program mode, ISO 100, a shutter speed of 1/250 second, focus distance of 35 cm, and zoom ratio of 2:1.

**Figure 1:**
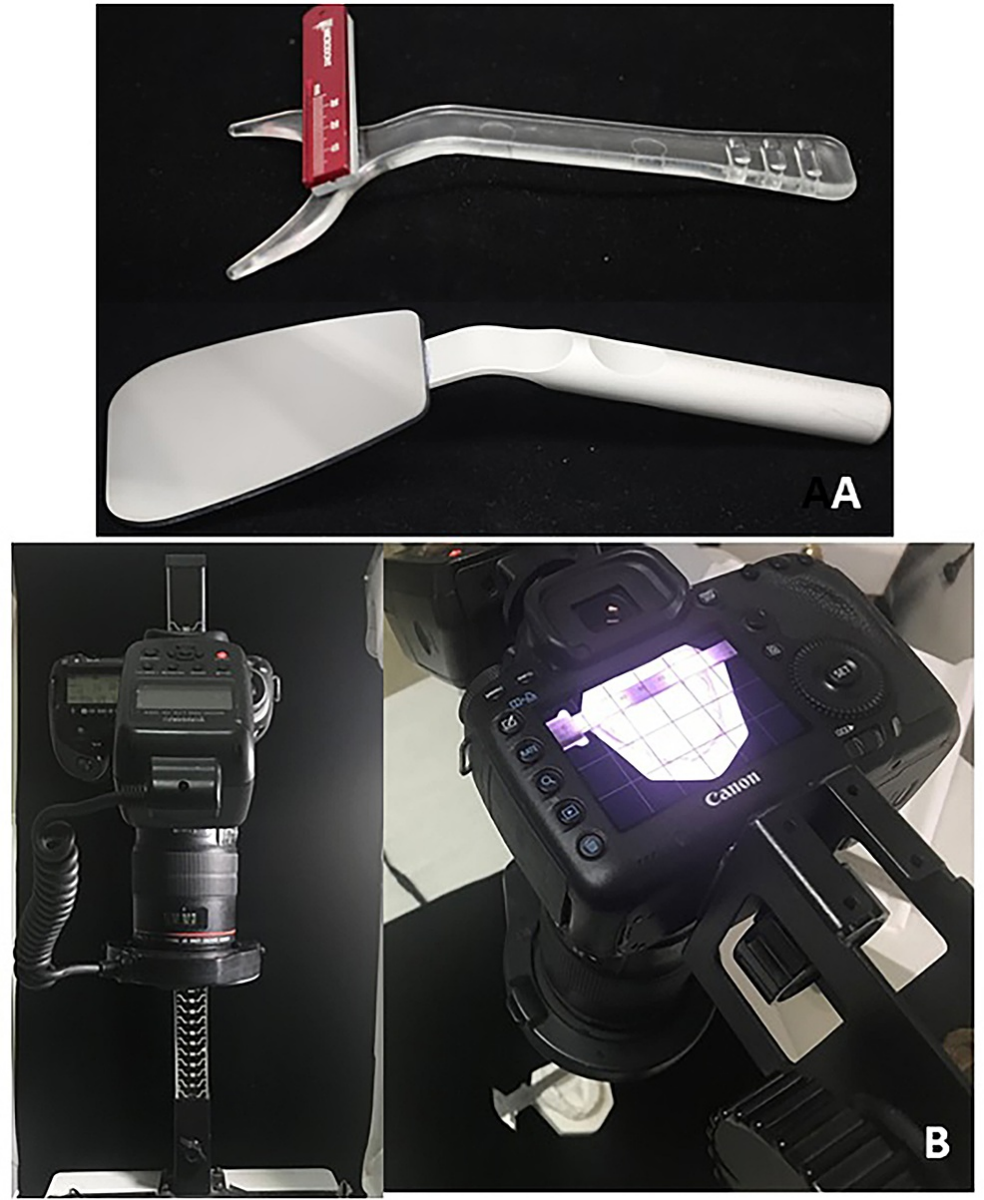
Materials used in this study. A: The employed lip retractor with a fixed ruler in addition to the occlusal mirror. B: The used camera is mounted on a stand.

**Figure 2:**
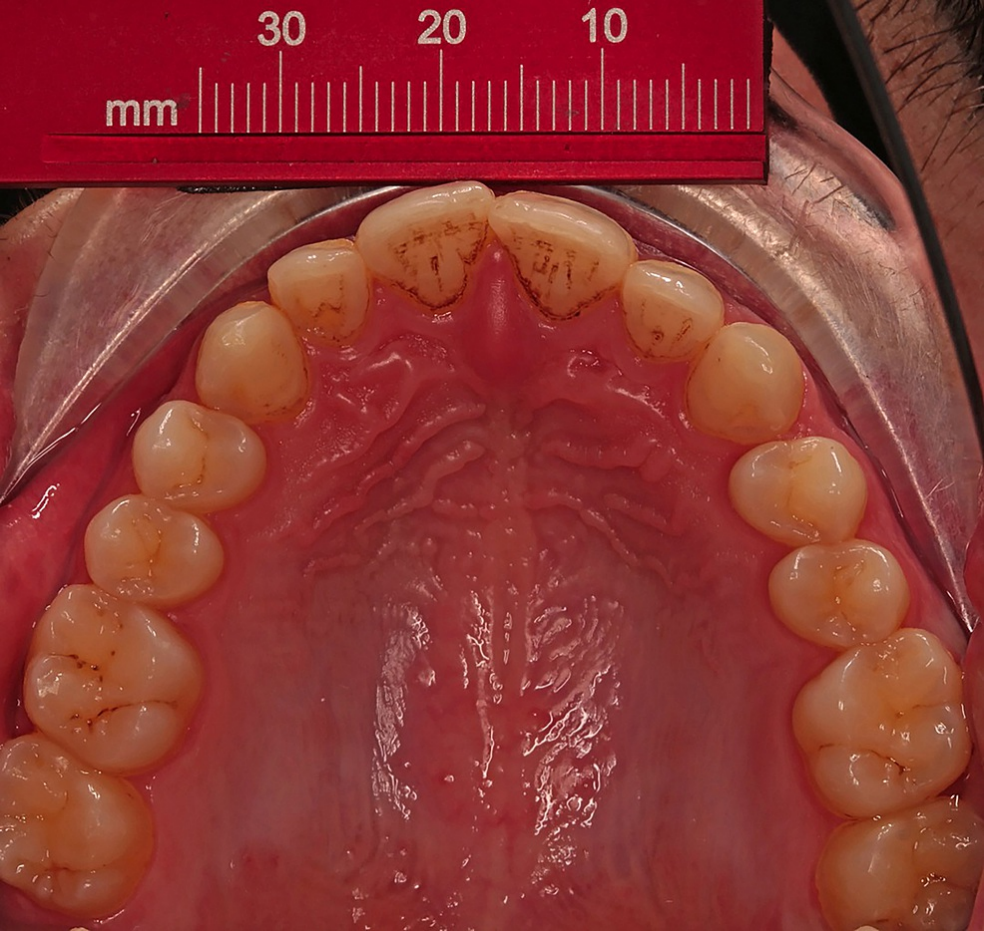
An intraoral photograph showing the embedded ruler for the calibration procedure.

Photographs were only accepted for inclusion in the current study when they showed the labial and lingual surfaces of the anterior teeth according to the clinical guide mentioned by Almasoud and Bearn [25]. A ruler was fixed by double-sided adhesive on a lip expander (Retract EEZ TM, Ortho Technology, West Columbia, SC) for calibration purposes and used as an occlusal mirror (Hahnenkratt photo mirror form 2, E. Hahnenkratt GmbH, Königsbach-Stein, Germany), as shown in Figures 1A, 2. Study models were photographed using a camera mounted on a stand (LPL 6000 Series Medium Format Enlargers, KHB Photografix, Welland, Ontario, Canada) with the same previous settings except for the distance that was set at 42 cm as shown in Figure 1B.

Validity of intraoral photographs and plaster models' photographs

Measurements of LII, the sum of teeth sizes (STS), basal bone length (BBL), TSALD, and Bolton's analysis were undertaken on (1) plaster models, (2) calibrated intraoral photographs of the dental arches, and (3) photographs of the same plaster models. Then the measurements made on photographs were compared against the gold standard values (i.e., plaster models' measurements).

Intra- and inter-observer reliability of measurements

All measurements were repeated after two weeks by the same examiner (A.O.) to determine intra-observer reliability and by another examiner (W.R.) to determine inter-observer reliability.

Reproducibility of the procedure of intraoral imaging

Another set of intraoral photographs was taken by the first examiner (A.O.) and the measurements were performed again to evaluate the reproducibility of the procedure of intraoral imaging. All measurements on photographs were recorded using Image-J software version 1.71 (developed by Werner Bailer, Austria University of Applied Sciences, Hagenberg, Austria). Measurements on study models were made using a dental manual caliper (Zuricher 130 mm, Medesy, Italy) with a precision of 0.1 mm.

Statistical analysis

IBM® SPSS® program version 20 (IBM Corp., Armonk, NY) was employed to test the correlation between measurements using intraclass correlation coefficient (ICC) and paired t-test to determine the differences.

## Results

Validity and reliability of measurements made on intraoral images

The validity and reliability of measurements made on intraoral photographs by examiners A and B are given in Tables 1, 2, respectively. All measurements showed high ICCs when compared with the gold standard readings and they were greater than 90%. For the first examiner, mean differences between measurements made on intraoral photographs versus the gold standard values were between 0.10 mm for TSALD values and 0.45 mm for the sum of teeth size. All variables showed a statistical significance except TSALD and overall Bolton value, whereas for the second examiner, the highest difference was 0.27 mm for partial Bolton value and the differences were not statistically significant.

**Table 1:** Validity and reliability of measurements made on intraoral photographs of the dental arches compared to those made on plaster models (examiner A). * P < 0.05, ** P < 0.01, and *** P < 0.001. ICC: intraclass correlation coefficient; SD: standard deviation; TSALD: tooth size-arch length discrepancy; LII: Little's Irregularity Index; BBL: basal bone length; STS: sum of teeth sizes.

Variable	ICC	Mean difference	SD	P-value	95% confidence interval	
STS	0.98	−0.45	0.91	0.011*	−0.70	−0.19
BBL	0.98	−0.39	0.40	0.002***	−0.50	−0.27
TSALD value	0.90	−0.10	0.95	0.652	−0.32	−0.20
LII	099	−0.16	0.35	0.002**	−0.25	−0.06
Partial Bolton	0.98	−0.24	0.51	0.002**	−0.46	−0.03
Overall Bolton	0.97	−0.40	0.56	0.691	−0.28	0.19

**Table 2:** Validity and reliability of measurements made on intraoral photographs of the dental arches compared to those made on plaster models (examiner B). ICC: intraclass correlation coefficient; SD: standard deviation; TSALD: tooth size-arch length discrepancy; LII: Little's Irregularity Index; BBL: basal bone length; STS: sum of teeth sizes.

Variable	ICC	Mean difference	SD	P-value	95% confidence interval	
STS	0.99	0.10	0.59	0.852	−0.15	0.18
BBL	0.99	0.12	0.32	0.385	−0.05	0.13
TSALD value	0.95	−0.16	0.65	0.792	−0.20	0.15
LII	0.99	−0.12	0.26	0.127	−0.13	0.16
Partial Bolton	0.92	−0.27	1.13	0.236	−0.74	0.19
Overall Bolton	0.94	−0.24	0.79	0.576	−0.42	0.24

Validity and reliability of measurements made on plaster models' images

The evaluation of measurements made on images taken of poured plaster models compared to the gold standard measurements is given in Table 3. A high level of agreement was found for all variables with ICCs more than 0.88 and mean differences ranging from 0.14 mm for the basal bone length to 0.48 mm for the sum of teeth size with no statistically significant differences for all variables except for the LII and the TSALD measurements (P < 0.001 and P = 0.026, respectively).

**Table 3:** Validity and reliability of measurements made on photographs of plaster models compared to those made on plaster models (examiner A). * P < 0.05 and *** P < 0.001. ICC: intraclass correlation coefficient; SD: standard deviation; TSALD: tooth size-arch length discrepancy; LII: Little's Irregularity Index; BBL: basal bone length; STS: sum of teeth sizes.

Variable	ICC	Mean difference	SD	P-value	95% confidence interval of mean difference	
STS	0.93	−0.48	1.81	0.063	−0.99	0.02
BBL	0.97	−0.14	1.19	0.382	−0.48	0.18
TSALD value	0.88	−0.33	1.04	0.026*	−0.62	−0.04
LII	0.99	−0.19	0.29	<0.001***	−0.28	−0.11
Partial Bolton	0.94	−0.28	0.96	0.155	−0.68	0.11
Overall Bolton	0.96	−0.20	0.66	0.510	−0.37	0.19

Intra- and inter-examiner reliability of the different measuring techniques

Intra-examiner reliability of measurements made on plaster models is given in Table 4. ICCs were greater than 0.90 for all measurements and the mean differences between repeated measurements ranged from 0.17 mm for LII to 0.33 for partial Bolton value with a significant difference found for the LII and TSALD (P < 0.001 for both variables).

**Table 4:** Intra-examiner reliability of measurements made on plaster models (examiner A). *** P < 0.001. ICC: intraclass correlation coefficient; SD: standard deviation; TSALD: tooth size-arch length discrepancy; LII: Little's Irregularity Index; BBL: basal bone length; STS: sum of teeth sizes.

Variable	ICC	Mean difference	SD	P-value	95% confidence interval of mean difference	
STS	0.90	−0.19	2.50	0.578	−0.89	0.50
BBL	0.91	0.24	2.36	0.463	−0.42	0.91
TSALD value	0.94	−0.27	0.66	<0.001***	−0.46	−0.08
LII	0.99	−0.17	0.24	<0.001***	−0.24	−0.11
Partial Bolton	0.91	−0.33	1.2	0.193	−0.85	0.18
Overall Bolton	0.96	−0.20	0.66	0.150	−0.48	0.07

The intra-examiner reliability of measurements made on intraoral images of dental arches showed a high level of agreement with ICCs being greater than 0.85, whereas the mean differences between the repeated measurements ranged from 0.04 mm for LII to 0.18 mm for the sum of teeth size. All these differences were not statistically significant as shown in Table 5. This indicated a high competency between the first and second measurements on the same intraoral photographs and high intra-examiner reliability.

**Table 5:** Intra-examiner reliability of measurements made on intraoral photographs of the dental arches (examiner A). ICC: intraclass correlation coefficient; SD: standard deviation; TSALD: tooth size-arch length discrepancy; LII: Little's Irregularity Index; BBL: basal bone length; STS: sum of teeth sizes.

Variable	ICC	Mean difference	SD	P-value	95% confidence interval	
STS	0.98	0.18	0.94	0.159	−0.07	0.45
BBL	0.98	0.07	0.89	0.543	−0.17	0.32
TSALD value	0.85	0.11	1.24	0.524	−0.23	0.46
LII	0.98	0.04	0.43	0.487	−0.08	0.16
Partial Bolton	0.99	0.12	0.41	0.947	−0.16	0.17
Overall Bolton	0.98	−0.10	0.36	0.173	−0.25	0.04

The analysis of inter-examiner reliability of plaster models’ measurements and those of intraoral photos showed high ICCs that ranged between 0.92 for partial Bolton ratio and 0.99 for STS and LII, indicating a high level of agreement between both examiners. The mean differences between the two examiners’ measurements when evaluating plaster models ranged from 0.08 mm for LII to 0.24 mm for partial Bolton ratio with no significant differences except for the sum of teeth size and LII (P = 0.041 and P < 0.001, respectively; Table 6). On the other hand, the mean differences when evaluating intraoral photographs ranged from 0.12 mm for overall Bolton ratio to 0.27 mm for TSALD with no significant differences observed for all variables (Table 7). This indicated a high consistency between the first and second examiners.

**Table 6:** Inter-examiner reliability of measurements made on plaster models (examiners A and B). * P < 0.05 and *** P < 0.001. ICC: intraclass correlation coefficient; SD: standard deviation; TSALD: tooth size-arch length discrepancy; LII: Little's Irregularity Index; BBL: basal bone length; STS: sum of teeth sizes.

Variable	ICC	Mean difference	SD	P-value	95% confidence interval	
STS	0.99	−0.10	0.35	0.041*	−0.20	<0.01
BBL	0.97	−0.20	0.21	0.338	−0.30	0.25
TSALD value	0.98	−0.12	0.38	0.174	−0.18	0.03
LII	0.99	−0.08	0.18	<0.001***	−0.13	−0.03
Partial Bolton	0.92	−0.24	1.09	0.271	−0.69	0.20
Overall Bolton	0.95	−0.22	0.78	0.926	−0.34	0.31

**Table 7:** Inter-examiner reliability of measurements made on intraoral photographs of the dental arches (examiners A and B). ICC: intraclass correlation coefficient; SD: standard deviation; TSALD: tooth size-arch length discrepancy; LII: Little's Irregularity Index; BBL: basal bone length; STS: sum of teeth sizes.

Variable	ICC	Mean difference	SD	P-value	95% confidence interval	
STS	0.98	0.21	0.99	0.124	−0.06	0.49
BBL	0.98	0.17	0.82	0.143	−0.06	0.40
TSALD value	0.88	0.27	1.13	0.778	−0.27	0.36
LII	0.98	0.19	0.46	0.150	−0.03	0.22
Partial Bolton	0.97	0.14	0.58	0.696	−0.19	0.28
Overall Bolton	0.97	0.12	0.53	0.635	−0.17	0.28

Reproducibility of measurements made on intraoral images of the dental arches

The evaluation of measurements made on the first set of intraoral images compared to the second set of the same dental arches showed a high level of agreement for all variables. ICCs were more than 0.96 and the mean differences between repeated measurements ranged from 0.03 mm for the basal bone length to 0.22 for the partial Bolton ratio with no statistically significant differences for all variables except for the LII (P = 0.018) as shown in Table 8.

**Table 8:** Reproducibility of measurements made on intraoral photographs of the dental arches at two different occasions. * P < 0.05. ICC: intraclass correlation coefficient; SD: standard deviation; TSALD: tooth size-arch length discrepancy; LII: Little's Irregularity Index; BBL: basal bone length; STS: sum of teeth sizes.

Variable	ICC	Mean difference	SD	P-value	95% confidence interval	
STS	0.99	−0.08	0.49	0.212	−0.22	0.05
BBL	0.99	−0.03	0.43	0.612	−0.15	0.09
TSALD value	0.98	−0.05	0.42	0.347	−0.17	0.06
LII	0.99	0.07	0.23	0.018*	0.01	0.14
Partial Bolton	0.99	−0.22	0.56	0.055	−0.005	0.46
Overall Bolton	0.96	−0.19	0.60	0.130	−0.06	0.44

## Discussion

When a scan or photocopy of the plaster model is properly taken, the produced photograph is expected to be perpendicular to the occlusal plane. This matter does not exist with the intraoral photography of the dental arches using hand-held cameras and may introduce errors of projection. The angulation at which the intraoral photograph is taken may vary so that a vertical component of the contact points displacement can be introduced to the measurement [25]. Additionally, photographs may also have errors of magnification, so the calibration process (correction of magnification) must be taken into account before any measurement is carried out on a digital photo.

The study model measurements were considered as the gold standard for testing photograph-based measurements validity. Indeed, manual measuring on study models may suffer from some inherent errors (e.g. vibrations and sometimes calipers cannot reach the exact interproximal contact point of a tooth when that tooth is in contact with other teeth) [4,26].

Differences between the evaluated two methods of measuring that were greater than 0.25 mm were considered clinically important for small measurements (e.g. LII) and the threshold of clinically significant differences was raised to 1 mm for those greater measurements in the orthodontic study model analyses (e.g. TSALD, Bolton ratios, and the sum of tooth size).

In contrast to the current study, previous papers [23-25] tested the validity of photographs when measuring LII only. Orthodontic diagnosis and treatment planning depends on several dental and alveolar analyses and the assessment of LII solely is not enough for decision-making and outcomes analysis in daily practice. Therefore, this study was accomplished to cover a wide spectrum of analyses that evaluate dental arches in the context of orthodontic evaluation.

Almasoud and Bearn used a millimeter ruler placed with wax onto the cusp tips of the premolars in the same level of the occlusal plane crossing the dental arch horizontally [25]. This ruler helped in correcting the magnification of intraoral photographs but was not practically easy to use since the applied wax may have not provided good and stable contact with the teeth as well as being cumbersome to the evaluated patients. Additionally, the fixation of the ruler at the middle of the dental arch prevented the ability of measurements in the posterior regions, so less chance to apply several dental arch analyses. Therefore, in the current trial, the ruler was fixed on the periphery of the lip retractor, enabling the operator to take photos without any additional time-wasting procedures.

Validity and reliability of measurements made on intraoral images

The differences in teeth sizes, basal bone length, LII, and partial Bolton measured on intraoral images by examiner A were statically significant when compared to those obtained from study models. However, the differences were equal to or less than 0.25 mm for the LII and 0.45 mm for the other variables, which appeared to be of no clinical importance. On the other hand, TSALD, overall Bolton value measured by examiner A, and all variables measured by examiner B did not show any statistically significant differences from those obtained from study models, indicating that all measurements on intraoral photographs were of a high, and sometimes a very good, level of agreement with those of plaster models. The current findings are similar to those of Almasoud and Bearn who evaluated only the LII measurement [25]. Nevertheless, the current 95% confidence intervals of mean differences were narrower than those of Almasoud and Bearn. This may be due to differences in the calibration method, camera and lens settings, or mirror reflecting properties that may have affected picture resolution.

Validity and reliability of measurements made on plaster models' images

The comparison between measurements made on plaster models and the corresponding images of these study models showed high ICCs, which were greater than 0.88, and the differences were less than 0.19 mm for LII and 0.48 mm for the other variables. Since the differences of LII and TSALD measurements were less than the aforementioned clinical thresholds, good validity and reliability could be inferred. These findings are consistent with those of Almasoud and Bearn, Mushtaq et al., and Tran et al., who employed digital images of study models in their assessments [23-25]. Tran et al. reported that assessment of LII in class II crowded mandibular arches was very reliable and valid; unfortunately, they presented only correlation coefficients without performing significance testing [24].

Intra- and inter-examiner reliability of the different measuring techniques

The analysis of intra-examiner reliability of measurements made on plaster models revealed high ICCs. On the other hand, significant differences smaller than 0.27 mm (and 0.17 mm for LII) were found between the repeated measurements of one examiner (i.e., examiner A) regarding LII and TSALD values but these differences could be neglected since they do not affect the clinical decision.

The intra-examiner reliability of measurements made on intraoral images of dental arches showed high ICCs and all obtained values were greater than 0.85. In addition, mean differences were not significantly different between the two sets of data. This indicated a high agreement between the first and second measurements and high intra-examiner reliability.

The analysis of inter-examiner reliability of plaster models’ measurements and those of intraoral photos showed high ICCs that ranged between 0.92 and 0.99. Significant differences with P-values less than 0.05 were found in teeth size and LII variables between the two examiners (i.e., examiner A and examiner B) on plaster models but these differences were less than 0.10 mm, which did not have any clinical importance. No significant differences were identified between the two examiners regarding measurements made on intraoral photographs. This indicated a high level of agreement between the first and second examiners when using the same intraoral photographs and high inter-examiner reliability. This is similar to the previous reports by Almasoud and Tran [24,25].

Actually, the intraoral photographs method was better than direct measurement on study models in terms of intra- and inter-examiner reliability, particularly for the TSALD analysis due to obtaining high ICC values. A similar conclusion was arrived at by Almasoud and Bearn regarding LII measurement. This can be attributed to the error introduced in manual measuring if any vibration occurs and secondly to the inability of calipers’ heads to rest at the exact interproximal contact points of neighboring teeth when they are in close contact or overlapped.

Reproducibility of measurements made on intraoral images

The comparison between results obtained from the first and second intraoral photographs showed a significant difference just for LII but the mean difference was lower than the aforementioned clinical threshold (i.e., 0.25 mm for LII).

In the light of the current results, measurements on intraoral photographs of the dental arches were very accurate, reliable, and repeatable when performing dental arch analysis. Additionally, they are suggested to replace study models and eliminate the need for repeated impressions during orthodontic treatment with fixed appliances in the context of prospective research projects.

## Conclusions

Measurements of LII, TSALD, and partial and overall Bolton analysis performed on intraoral images of the dental arches, and images of study models were valid when compared to the gold standard measurements. Additionally, all the aforementioned measurements performed on intraoral photographs and images of study models were reliable regarding the intra- and inter-observer reliability, and the whole procedure of capturing the dental arches was found reproducible.
